# Identification of potential therapeutic targets for COVID-19 through a structural-based similarity approach between SARS-CoV-2 and its human host proteins

**DOI:** 10.3389/fgene.2024.1292280

**Published:** 2024-02-02

**Authors:** Alvea Tasneem, Armiya Sultan, Prithvi Singh, Hridoy R. Bairagya, Hassan Hussain Almasoudi, Abdulfattah Yahya M. Alhazmi, Abdulkarim S. Binshaya, Mohammed Ageeli Hakami, Bader S. Alotaibi, Alaa Abdulaziz Eisa, Abdulaziz Saleh I. Alolaiqy, Mohammad Raghibul Hasan, Kapil Dev, Ravins Dohare

**Affiliations:** ^1^ Centre for Interdisciplinary Research in Basic Sciences, Jamia Millia Islamia, New Delhi, India; ^2^ Department of Biotechnology, Faculty of Natural Sciences, Jamia Millia Islamia, New Delhi, India; ^3^ Department of Bioinformatics, Maulana Abul Kalam Azad University of Technology, Haringhata, West Bengal, India; ^4^ Department of Clinical Laboratory Sciences, College of Applied Medical Sciences, Najran University, Najran, Saudi Arabia; ^5^ Clinical Pharmacy Department, Umm Al-Qura University, Makkah, Saudi Arabia; ^6^ Department of Medical Laboratory Sciences, College of Applied Medical Sciences, Prince Sattam Bin Abdulaziz University, Alkharj, Saudi Arabia; ^7^ Department of Clinical Laboratory Sciences, College of Applied Medical Sciences, Al- Quwayiyah, Shaqra University, Riyadh, Saudi Arabia; ^8^ Department of Medical Laboratory Technology, College of Applied Medical Sciences, Taibah University, Medina, Saudi Arabia; ^9^ Forensic Medical Services Center, Ministry of Health, Qassim, Saudi Arabia

**Keywords:** COVID-19, virus–host target proteins, structural-based similarity, PPI network, SARS-CoV-2

## Abstract

**Background:** The COVID-19 pandemic caused by SARS-CoV-2 has led to millions of deaths worldwide, and vaccination efficacy has been decreasing with each lineage, necessitating the need for alternative antiviral therapies. Predicting host–virus protein–protein interactions (HV-PPIs) is essential for identifying potential host-targeting drug targets against SARS-CoV-2 infection.

**Objective:** This study aims to identify therapeutic target proteins in humans that could act as virus–host-targeting drug targets against SARS-CoV-2 and study their interaction against antiviral inhibitors.

**Methods:** A structure-based similarity approach was used to predict human proteins similar to SARS-CoV-2 (“hCoV-2”), followed by identifying PPIs between hCoV-2 and its target human proteins. Overlapping genes were identified between the protein-coding genes of the target and COVID-19-infected patient’s mRNA expression data. Pathway and Gene Ontology (GO) term analyses, the construction of PPI networks, and the detection of hub gene modules were performed. Structure-based virtual screening with antiviral compounds was performed to identify potential hits against target gene-encoded protein.

**Results:** This study predicted 19,051 unique target human proteins that interact with hCoV-2, and compared to the microarray dataset, 1,120 target and infected group differentially expressed genes (TIG-DEGs) were identified. The significant pathway and GO enrichment analyses revealed the involvement of these genes in several biological processes and molecular functions. PPI network analysis identified a significant hub gene with maximum neighboring partners. Virtual screening analysis identified three potential antiviral compounds against the target gene-encoded protein.

**Conclusion:** This study provides potential targets for host-targeting drug development against SARS-CoV-2 infection, and further experimental validation of the target protein is required for pharmaceutical intervention.

## 1 Introduction

At present, severe acute respiratory syndrome coronavirus 2 (SARS-CoV-2) has spread rapidly, infecting ∼769 million people globally, including ∼6 million reported deaths as of 16 August 2023 (https://covid19.who.int/). Treatment options for COVID-19 disease include oral medications and intravenous therapy for mild and severe patients, while vaccines are primarily administered as a preventive measure (Ali et al., 2020; Morse et al., 2020; Martín-Sánchez et al., 2023). The use of vaccines initially curbed the SARS-CoV-2 pandemic. However, with the emergence of the Omicron variant (Fan et al., 2022), the efficacy of vaccination against the infection has decreased with each viral lineage. Antiviral drug candidates have provided effective treatment against viral infections, yet their use may be limited by the rapid evolutionary rate of viruses and the possibility of resistance emerging (Dai et al., 2020; Dolgin, 2021; Burki, 2022). Therefore, it is essential to continue the development of alternative antiviral therapies to combat the ongoing threat of emerging viral infections. Host-directed therapy (HDT) has gained momentum in the past decade (Tripathi et al., 2021). Host-targeting drugs with a low evolutionary divergence compared to viruses may avoid treatment failure. Similarities in motif sequence or protein structures between the virus and host proteins can provide clues for predicting virus–human protein–protein interactions (PPIs) (Mariano and Wuchty, 2017; Liu X. et al., 2021). Thus, investigating these PPIs between a virus and its human host could contribute to discovering the viruses’ potential human host target proteins (Liu X. et al., 2021).

Although finding intraspecies PPIs is common, predicting PPIs among different species is rare; therefore, this study may allow researchers to understand not only how a pathogenic protein interacts with its host on a molecular level but also how such interactions function in a more extensive cellular network (Lee et al., 2008). In this study, to elucidate the host–virus protein–protein interactions (HV-PPIs), a structure-based interaction approach was implemented to identify PPIs among human proteins (HPs) with known interactions that are structurally similar to viral proteins (Franzosa and Xia, 2011; Keskin et al., 2016; Rajasekharan et al., 2016). The primary limitation of using a structure-based interaction approach is the inadequate availability of three-dimensional structures and protein interaction of either pathogens or their host (Mariano and Wuchty, 2017). However, several studies have used a structural similarity approach to identify human–pathogen PPIs. Davis et al. were the first to demonstrate the use of 3D structural homology for predicting interspecies PPIs. They scanned the genomes of the host and pathogen for proteins similar to known putative interactions, using structural information, and filtered the remaining interactions based on the biological context for various human pathogens (Davis et al., 2007).

Similarly, Doolittle and Gomez (2011) predicted 502 interactions between HIV and human proteins by using the structure similarity between nine HIV-1 proteins and human proteins with known interactions, functional data from RNAi studies, and cellular component annotation from the Gene Ontology database (Doolittle and Gomez, 2011). Sagar et al. (2021) used a structural similarity approach to identify host–pathogen interactions between 10 Zika virus (ZIKV) proteins and their two distinct hosts, human and *D. melanogaster* (Sagar et al., 2021). Based on previous studies, it has been proposed that if there are pairs of structure-similar virus–host proteins with known interacting host proteins, the target host proteins may also interact with the viral proteins (Sagar et al., 2021). Furthermore, these target host proteins could either enhance the function of the virus or have an inhibitory effect. Hence, exploring the common pathways, molecular functions, biological processes, cellular compartments, and other properties of the host proteins interacting with the virus can help inhibit infectious disease. These virus–host-targeting proteins may act as a potential drug target for the design of antiviral inhibitors.

The present study used a structure-based similarity approach to predict human host proteins similar to SARS-CoV-2 (“hCoV-2”), followed by the identification of PPIs between hCoV-2 and its target human proteins from different protein interaction databases (referred to as “T_hCoV-2”). Additionally, overlapping genes were identified between the protein-coding genes of T_hCoV-2 and COVID-19-infected patient’s messenger RNA (mRNA) expression data obtained from the NCBI-GEO. The overlapped genes between target and infected groups (TIG-DEGs) were further processed to identify differentially expressed genes (DEGs). Pathway and Gene Ontology (GO) enrichment analyses, construction of PPI networks, and detection of hub gene modules for TIG-DEGs were performed. Last, structure-based virtual screening with an antiviral compound database was performed to identify potential hits against the target gene-encoded protein. The vision of the present study was to identify target proteins in humans that may act as host-targeting drug targets against SARS-CoV-2 infection. Further experimental validation of the target protein is required for pharmaceutical intervention against SARS-CoV-2.

## 2 Materials and methods

### 2.1 Viral protein structure collection

The experimentally determined three-dimensional structures of SARS-CoV-2 [envelope protein (PDB ID: 7K3G), main protease (PDB ID: 5R7Y), spike glycoproteins (PDB ID: 6VSB, 6VXX), HR2 domain (PDB ID: 6LVN, 6M1V), NSP2 (PDB ID: 7MSW, 7MSX), NSP3 (PDB ID: 6W02, 6W6Y, 6W9C), NSP7 (PDB ID: 6WTC), NSP8 (6M5I), NSP9 (PDB ID: 6W9Q, 6W4B, 6WCI), NSP12 (PDB ID: 6XEZ, 6M71), NSP13 (PDB ID: 5RL6), NSP14 (PDB ID: 7DIY), NSP15 (PDB ID: 6VWW, 7K0R), NSP16 (PDB ID: 6W4H), ORF3a (PDB ID: 6XDC), ORF7a (PDB ID: 6W37), and ORF8 (PDB ID: 7JTL)] were obtained from the RCSB Database (https://www.rcsb.org/) (Rose et al., 2017). The remaining coronavirus structures [membrane protein (M), NSP4, and ORF6] that are not available in the Protein Data Bank (PDB) were modeled using Contact-guided Iterative Threading ASSEmbly Refinement (C-I-TASSER) (https://zhanggroup.org/C-I-TASSER/), an extended version of I-TASSER for high-accuracy protein structure and function predictions (Zheng et al., 2021). The modeled structures of M protein, NSP4, and ORF6 were verified using the Structural Analysis Verification Server (SAVES) program (https://saves.mbi.ucla.edu/) and protein structure analysis (ProSA)-web program (https://prosa.services.came.sbg.ac.at/prosa.php?pdbCode=1zyb) (Wiederstein and Sippl, 2007). The structural quality (PROCHECK) (Laskowski et al., 1993), non-bonded interactions (ERRAT) (Colovos and Yeates, 1993), and energy profile or Z-score (ProSA using molecular mechanics force field) were thoroughly checked and calibrated. The Z-scores obtained for the M protein, NSP4, and ORF6 were close to the related native conformations regarding their residue length, as determined by X-ray and NMR structures available in the RCSB database. Additionally, nearly 83%–88% of residues in these respective modeled structures were found to occupy favorable allowed regions in the Ramachandran plot (Hooft et al., 1997). It indicates that the modeled structures are high quality and likely biologically relevant.

### 2.2 Identification of structurally similar proteins among SARS-CoV-2 and its host

The structurally similar human proteins of SARS-CoV-2 were obtained by submitting the PDB IDs of viral protein structures (either known or C-I-TASSER-generated) to the Dali webserver (http://ekhidna2.biocenter.helsinki.fi/dali/) (Holm, 2022). In this study, only the *Homo sapiens* proteins with structural similarity score (Z-score) ≥ 2 were considered structurally similar proteins of the respective SARS-CoV-2 protein and are referred to as “hCoV-2” proteins. The PDB codes of hCoV-2 obtained from Dali were mapped to their corresponding UniProt ID and gene names by the UniProt Retrieve/ID mapping tool (https://www.uniprot.org/id-mapping) (Pundir et al., 2016).

### 2.3 Refinement of predictions

The Dali database may contain multiple structures of the same proteins in the PDB, which could result in repetitive interaction predictions. This issue can arise with certain SARS-CoV-2 proteins with multiple PDB structures, leading to repeating similar predictions. Hence, duplicate PDB IDs were removed before converting them to UniProt accession ID and gene names for target prediction. In addition, multiple hCoV-2 proteins (human proteins structurally similar to SARS-CoV-2) can have common cellular partners. Among these, unique pairs of interactions between human UniProt accession and SARS-CoV-2 proteins were considered.

### 2.4 Prediction of SARS-CoV-2–host protein interactions

Human endogenous protein interactors of SARS-CoV-2 were identified by obtaining the target protein interactors of hCoV-2 during various cellular processes. These cellular partners of hCoV-2 were obtained from HPRD (https://www.hprd.org/), BioGRID (https://thebiogrid.org/), HIPPIE (http://cbdm-01.zdv.uni-mainz.de/∼mschaefer/hippie/), and MINT (https://mint.bio.uniroma2.it/) databases and are referred to as T_hCoV-2 (target protein interactors of hCOV-2) (Baolin et al., 2007; Licata et al., 2012; Alanis-Lobato et al., 2017; Oughtred et al., 2019). These datasets are from literature-curated interactions established through *in vitro* and/or *in vivo* methods among human proteins. These cellular proteins, known to interact with hCoV-2 proteins, are presumed to interact with SARS-CoV-2 proteins due to their structural similarities with hCoV-2 proteins.

### 2.5 Comparison with the pre-processed dataset and differentially expressed gene analysis

The target protein interactors of hCoV-2, referred to as T_hCoV-2, of each of the SARS-CoV-2 proteins, were merged into one dataset corresponding to their HUGO Gene Nomenclature Committee (HGNC) symbols (https://www.genenames.org/) by eliminating the duplicate gene IDs to avoid redundancy. Pre-processed data corresponding to COVID-19 mRNA expression profiling were obtained from the NCBI-GEO (https://www.ncbi.nlm.nih.gov/geo/) (Jha et al., 2022). The unique target genes of T_hCoV-2 are then compared with the COVID-19 patient gene sample dataset from which only the overlapping genes between targets and infected datasets were considered for further DEG analyses.

To identify DEGs between T-hCoV-2 and COVID-19 samples, a two-sample statistical *t-*test was used, followed by obtaining their log_2_ (fold change) and Benjamin–Hochberg (BH) p−value through the limma R package (Ritchie et al., 2015). Genes with BH *p <* 0.05 and |log_2_ (FC) | > 2 were considered DEGs. DEGS with log_2_ (FC) > 2 and log_2_ (FC) < −2 were categorized as up- and downregulated, respectively. The DEGs that overlapped between targets and infected groups were referred to as TIG-DEGs.

### 2.6 Pathway, Gene Ontology terms, and PPI network construction of TIG-DEGs

Pathway and GO term enrichment data for TIG-DEGs were compiled using various libraries, *i.e.*, Kyoto Encyclopedia of genes and genomes (KEGG) and GO-biological process (BP), GO-molecular function (MF), and GO-cellular compartment (CC) terms available within the Enrichr database (https://maayanlab.cloud/Enrichr/) (Kuleshov et al., 2016). All the pathways and GO terms corresponding to *p* < 0.01 were determined as statistically significant.

The genes corresponding to the enrichment analysis were then merged into one dataset (eliminating duplicate gene names) and subjected to PPI network construction using the STRING v11.5 web-based tool (https://string-db.org/) (Szklarczyk et al., 2021). The construction of this PPI network was based on the highest confidence score >0.9, and it was visualized using Cytoscape v3.9.1 (Shannon et al., 2003). The topological properties of the PPI network were analyzed using the NetworkAnalyzer plugin in Cytoscape. Furthermore, the Molecular Complex Detection (MCODE) plugin was used to identify highly correlated gene clusters/modules within the PPI network. The parameters set in MCODE for cluster detection were as follows: “degree cutoff = 2,” “node score cutoff = 0.2,” “k-score = 2,” “max. depth = 100,” and “cut style = haircut.” The top-scoring genes in the PPI cluster were considered the hub genes.

### 2.7 Target identification and preparation for molecular docking

The *RPS3* gene has the most significant interaction partners of the top-scoring PPI cluster genes. Ribosomal protein subunit 3 (RPS3) is a protein-coding gene involved in the viral mRNA translation pathway. Since the viruses exploited the ribosomal proteins and the ribosomal biogenic processes to facilitate their replication, the RPs have been considered effective targets for developing antiviral agents (Dong et al., 2021). The three-dimensional electron microscopy structure of the human ribosomal subunit, PDB ID: 6ZLW (resolution: 2.60 Å), bounded to the SARS-CoV-2 NSP1 protein, was downloaded from the Protein Data Bank (https://www.rcsb.org/) (Rose et al., 2017). The two α-helices of NSP1 (residues: 154–179) of SARS-CoV-2 directly interact with RPS3, RPS2, and the phosphate backbone of rRNA helix 18 (h18) of 40S ribosomal protein. This interaction with RPS3, RPS2, and h18 rigidly anchors NSP1, obstructing the mRNA entry channel. Therefore, for this study, we took a subcomplex comprising RPS3, RPS2, and h18 as the targets for the molecular docking studies.

### 2.8 Virtual screening and ligand structure preparation

An *in silico* high-throughput virtual screening of known therapeutic COVID-19 drugs from the PubChem library was obtained (https://pubchem.ncbi.nlm.nih.gov/#query=covid-19) (Kim et al., 2019). The total search results were filtered thrice for final docking studies. Following are the filters for the selection of final screened compounds: filter 1, database filters (molecular weights should be from 100 to 500 g/mol, rotatable bond count from 0 to 7, h-bond acceptor count from 0 to 10, the polar area from 0 to 150 Å^2^, and XlogP from −6.3 to 5); filter 2, calculation of physicochemical properties (no Lipinski’s rule violations, no lead likeness violations, and high GI absorption) using the SwissADME web server (http://www.swissadme.ch/) (Daina et al., 2017); and filter 3, calculation of pharmacokinetics and toxicological properties (cLogP, solubility, TPSA, drug-likeness, drug score, mutagenic, tumorigenic, irritant, and reproductive effective) using the Osiris Property Explorer (Sander, 2001).

The 3D structures of the final screened compounds were generated using the OpenBabel program (O Boyle et al., 2011). The 2D SDF files were used as input files for OpenBabel for converting them to 3D structures, followed by energy minimization in MMFF94 force field (Halgren, 1996). Finally, all the structures of ligands were converted to the PDBQT format with appropriate rotatable bonds.

### 2.9 Molecular docking

The PDBQT file of the subcomplex receptor was prepared using Kollman united atom charges for molecular docking study using AutoDockTools v 1.5.6 (Singh and Kollman, 1984; Morris et al., 2009). As the subcomplex receptor contained two proteins and one nucleic acid, the tentative binding site for the inhibitor is unknown. Hence, a blind docking study using AutoDock Vina (v1.1.2) was performed (Trott and Olson, 2009). Grid point spacing was set at 1 Å, grid point 56 was taken in each direction of the grid box, and the concerned box was centered at the subcomplex receptor. The ligands bind to two probable sites of the subcomplex receptor—one at the rRNA h18 (ligand-binding site-I) and another at the RPS3 protein (II). The best three conformations of each ligand at two binding locations were identified after cluster analysis according to their lowest binding energy. The receptor–ligand hydrogen bond interactions were analyzed using the Swiss PDB viewer (Guex and Peitsch, 1997) and visualized using PyMOL v 2.5 (the PyMOL Molecular Graphics System, Version 1.2r3pre, Schrödinger, LLC).

## 3 Result

### 3.1 Identification of structurally similar human proteins of SARS-CoV-2 and hCoV-2 host PPIs

We used the Dali Lite webserver to identify human host proteins that exhibit structural similarity to the proteins of SARS-CoV-2. Twenty SARS-CoV-2 proteins were selected for this study (17 proteins from known sources, i.e., available via the RCSB PDB, and 3 proteins predicted or modeled using the C-I-TASSER program) (Table 1). The viral protein structures were then submitted to the Dali webserver to compare 3D structural coordinates of virus and human host proteins. To make the study more comprehensive and avoid redundancy, all the structurally similar host proteins for each viral protein were merged into one dataset, and duplicates were removed. For example, for NSP3 of SARS-CoV-2, the human proteins structurally similar to these three PDB IDs: 6W02, 6W6Y, and 6W9C were merged into one column, and duplicate human PDB IDs were then removed. A similar protocol was followed for the other viral proteins, with more than one PDB ID obtained for this study. It resulted in the identification of 6,116 human proteins (hCoV-2) similar to 20 SARS-CoV-2 proteins (Table 2 and Supplementary Table S1).

**TABLE 1:** List of 20 SARS-CoV-2 proteins selected for identifying structurally similar human host proteins.

S. no.	SARS-CoV-2 protein	Chain	Method	Sequence length	Resolution (Å)
1	**Envelope protein**				
	7K3G	A	NMR	31	NA*
2	**NSP2**				
	7MSW	A	EM	638	3.76
	7MSX	A	EM	638	3.15
3	**Papain-like protease (NSP3)**				
	6W02	A	X-ray	170	1.5
	6W6Y	A	X-ray	170	1.45
	6W9C	A	X-ray	317	2.7
4	**Main protease (NSP5)**				
	6LU7	A	X-ray	306	2.16
5	**NSP7**				
	6WTC-A	A	X-ray	86	1.85
6	**NSP8**				
	6M5I-A	A	X-ray	198	2.5
7	**NSP9**				
	6W9Q-A	A	X-ray	133	2.05
	6W4B-A	A	X-ray	117	2.95
	6WC1-A	A	X-ray	116	2.4
8	**RNA-dependent RNA polymerase (NSP12)**				
	6XEZ-A	A	EM	932	3.5
	6M71-A	A	EM	942	2.9
9	**Helicase (NSP13)**				
	5RL6	A	X-ray	601	1.92
10	**NSP14**				
	7DIY	B	X-ray	294	2.69
11	**NSP15**				
	6VWW	A	X-ray	370	2.2
	7K0R	A	EM	362	3.3
12	**NSP16**				
	6W4H	A	X-ray	301	1.8
13	**ORF3a**				
	6XDC	A	EM	284	2.9
14	**ORF7a**				
	6W37	A	X-ray	67	2.9
15	**ORF8**				
	7JTL	A	X-ray	107	2.04
16	**S2 subunit**				
	6LVN	A	X-ray	36	2.47
	6M1V	A	X-ray	119	1.5
17	**Spike protein**				
	6VSB	A	EM	1288	3.46
Modeled Structures using C-I-TASSER (predicted)					
		***Sequence ID***	***Sequence Length***	Estimate TM-score**	
18	**Membrane protein**	QHD43419	222	0.59	
19	**NSP4**	QHD43415.4	500	0.61	
20	**ORF6**	QHD43420	61	0.50	

**TABLE 2:** Number of hCoV-2 structurally similar proteins (known and predicted) to the SARS-CoV-2 proteins and its unique host interactors known as T_hCoV-2 proteins.

S. no.	SARS-CoV-2 protein	Structure type	Number of hCoV-2 similar proteins	Targeted T_hCoV-2 proteins
1	Envelope protein	Known	86	5863
2	NSP2	Known	371	1158
3	Papain-like protease (NSP3)	Known	83	342
4	Main protease (NSP5)	Known	67	204
5	NSP7	Known	393	473
6	NSP8	Known	576	513
7	NSP9	Known	76	90
8	RNA-dependent RNA polymerase (NSP12)	Known	06	04
9	Helicase (NSP13)	Known	466	257
10	NSP14	Known	17	10
11	NSP15	Known	30	33
12	NSP16	Known	60	34
13	ORF3a	Known	266	67
14	ORF7a	Known	678	825
15	ORF8	Known	628	41
16	S2 subunit	Known	995	558
17	Spike protein	Known	23	12
18	Membrane protein	Predicted	532	8167
19	NSP4	Predicted	08	15
20	ORF6	Predicted	755	385
	**Total**		6,116	19,051

Afterward, we identified all possible interacting partners of hCoV-2 proteins of the 20 proteins of SARS-CoV-2 from four different databases (HPRD, MINT, HIPPIE, and BioGRID). The target protein interactors of hCoV-2 from each database corresponding to 165,051 genes were merged into one unique dataset without duplicates. A total of 19,051 unique interactors were identified for these 20 SARS-CoV-2 proteins. These unique interactors are termed T_hCoV-2 proteins (Table 2 and Supplementary Table S2).

### 3.2 Comparison with the pre-processed dataset and differentially expressed gene analysis

**FIGURE 1:**
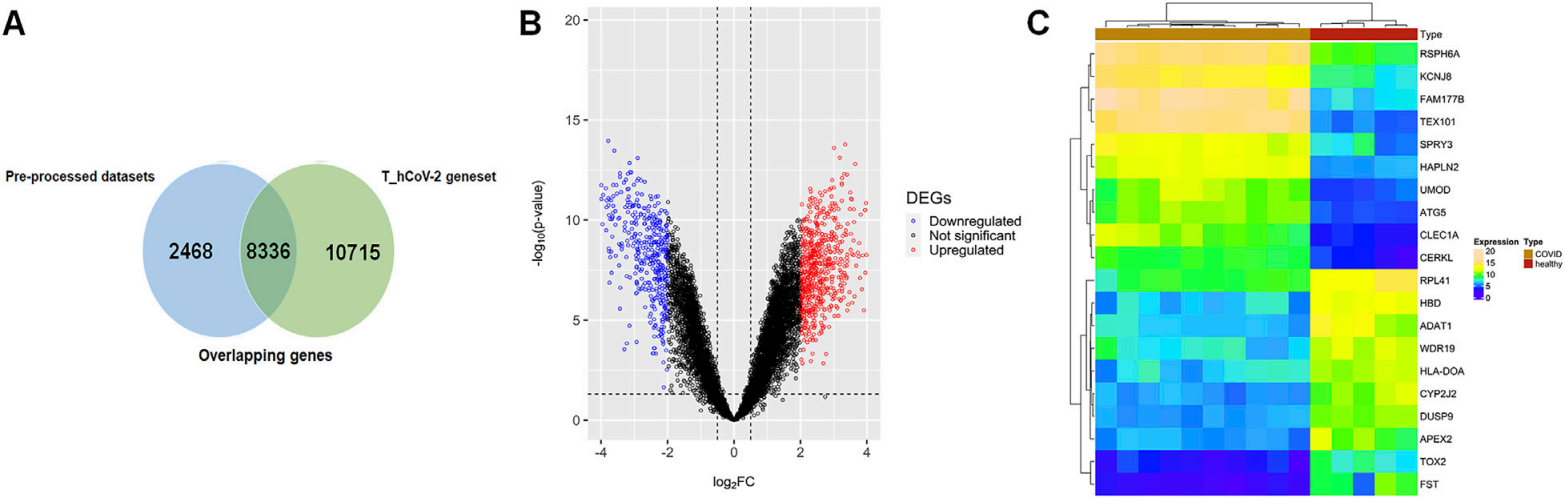
**(A)** Venn plot showing overlapping of pre-processed COVID-19 datasets across the T_hCoV-2 geneset. The blue-colored areas represent genesets pertaining to mild and severe infected COVID-19 microarray data, and green-colored areas represent genesets of T_hCOV-2. **(B)** Volcano plots show the distribution of significant (colored plots) and non-significant (black-colored points) genes in the microarray group. **(C)** Annotation heatmap showing the expression distribution of top 10 down- and upregulated DEGs across overlapping genes of COVID-19 and T_hCoV-2 datasets.

The pre-processed SARS-CoV-2 mRNA expression profile (GSE164805) datasets comprised five controls, and 10 COVID-19 (five mild and five severe) samples were compared with the T_hCoV-2 genes. The overlapping genes between targets and mRNA datasets amounted to 8,336 genes (Figure 1A). These genes were then differentially expressed corresponding to p−value <0.05 and $\left|\log_{2}\left(\text{fold change}\right)\right|>2$ . The DEGs overlapping between targets and infection severity groups were considered TIG-DEGs, which amount to 1,120 unique genes**.** Amongst these genes, 663 and 457 were up- and downregulated, respectively (Figure 1B). The most highly up- and downregulated DEGs were HBD ${\left[\log\right.}_{2}\left(FC\right)\left.=6.00\right]$ and TEX101 ${\left[\log\right.}_{2}\left(FC\right)\left.=-10.34\right]$ , respectively (Figure 1C).

### 3.3 Pathway and Gene Ontology enrichment analyses

**FIGURE 2:**
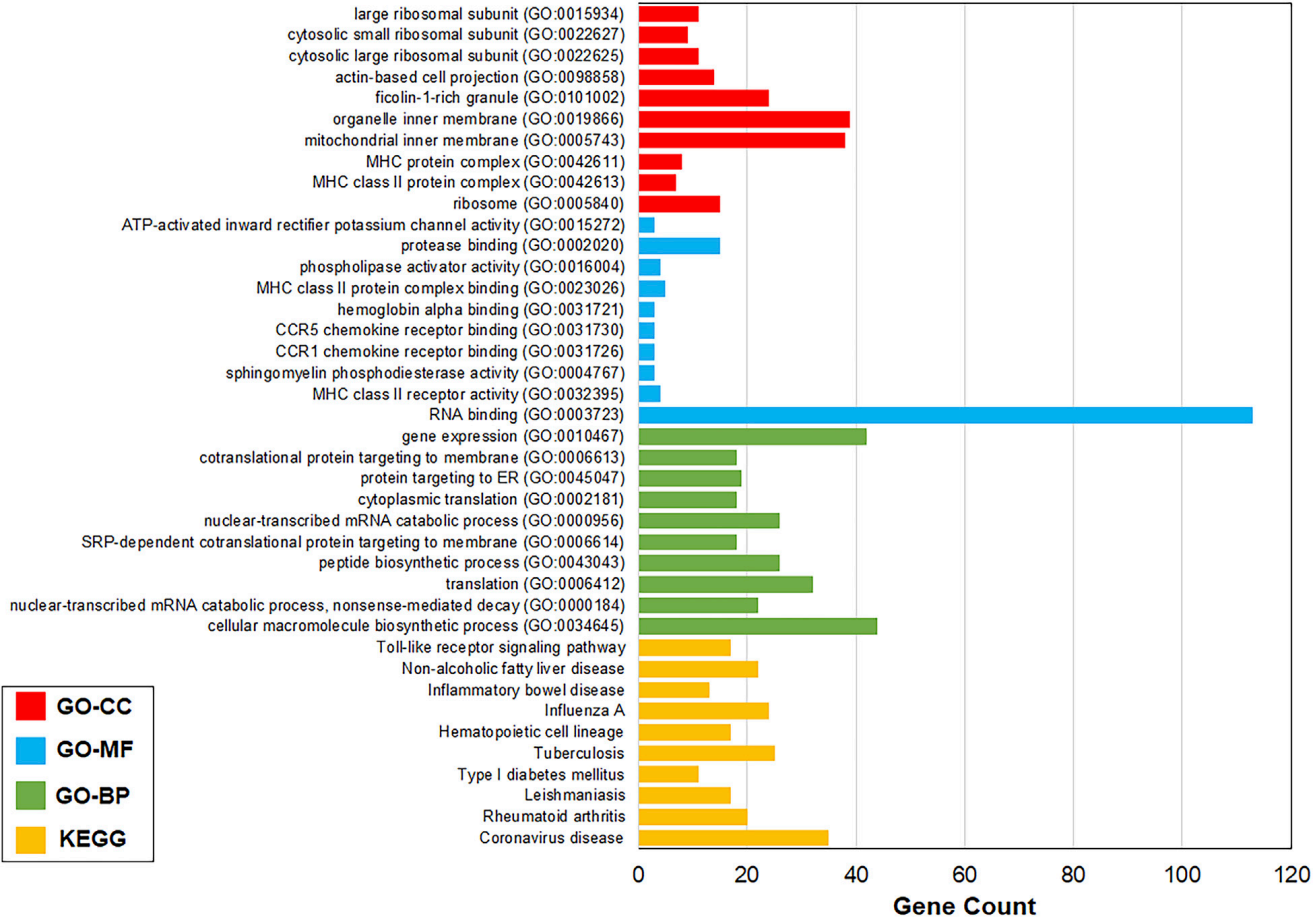
Top 10 most significant pathways and GO terms. Most significant pathways and GO-BP, GO-MF, and GO-CC terms were coronavirus disease ( p−value = 8.49 × 10^−8^), cellular macromolecule biosynthetic process ( p−value = 1.88 × 10^−8^), RNA binding ( p−value = 5.64 × 10^−5^), and ribosome ( p−value = 1.18 × 10^−6^). KEGG, Kyoto Encyclopedia of Genes and Genomes; GO, Gene Ontology; BP, biological process; MF, molecular function; and CC, cellular compartment.

All the 1,120 TIG-DEGs participated in the Gene Ontology and pathway enrichment analyses. All the significant pathways and GO-BP, GO-MF, and GO-CC terms ( p−value<0.01 ) are shown in Supplementary Table S3. The most significant pathways and GO-BP, GO-MF, and GO-CC terms were coronavirus disease ( $p-\text{value}=8.49\times 10^{-08}$ ), cellular macromolecule biosynthetic process ( $p-\text{value}=1.88\times 10^{-08}$ ), RNA binding ( $p-\text{value}=5.64\times 10^{-05}$ ), and ribosome ( $p-\text{value}=1.18\times 10^{-06}$ ) (Supplementary Table S3). For a more comprehensive representation, only the top 10 most significant pathways and GO terms are shown in Figure 2.

### 3.4 PPI network analysis of TIG-DEGs

**FIGURE 3:**
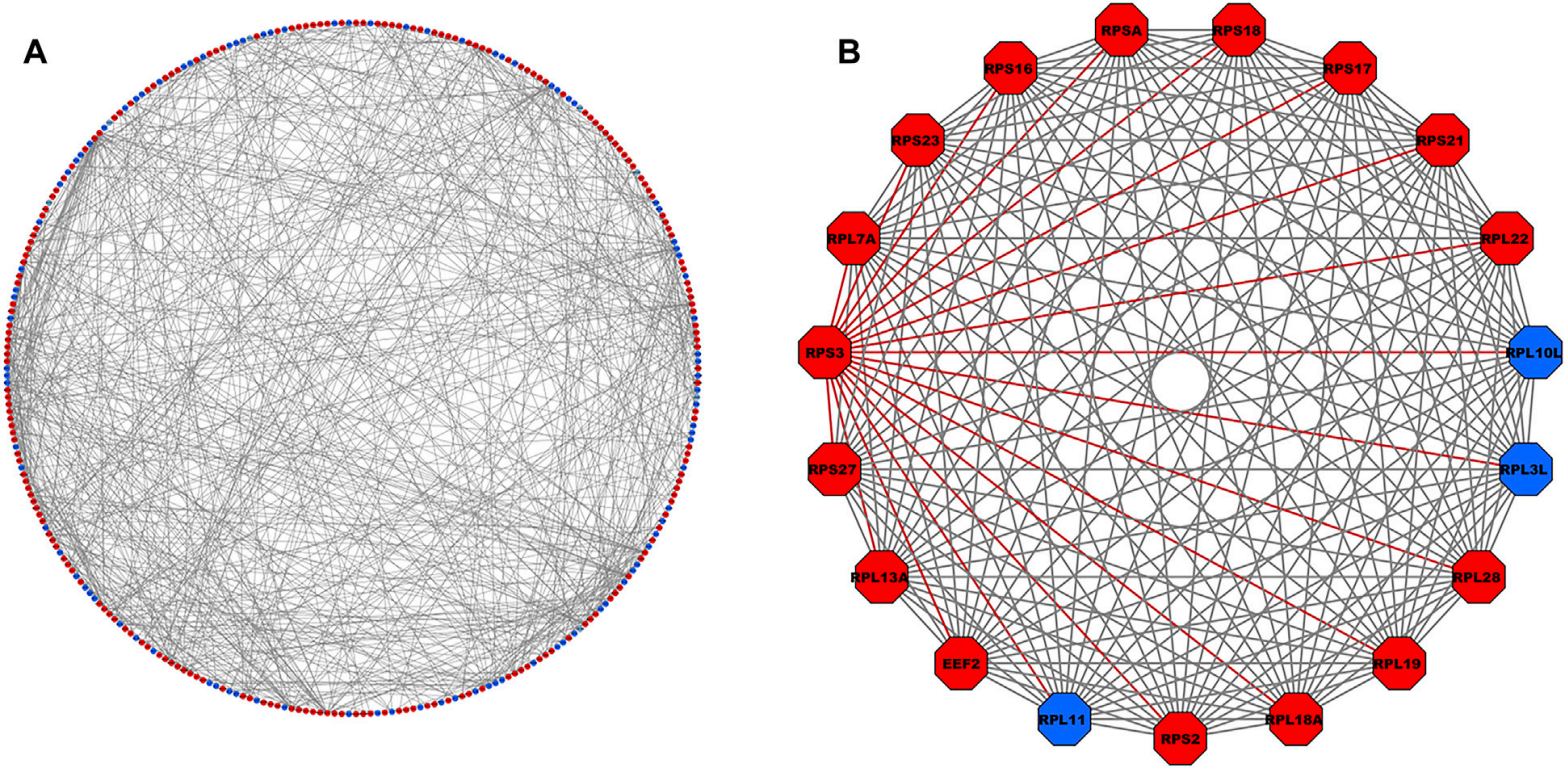
**(A)** PPI network comprising 302 nodes and 688 interacting edges constructed using the STRING database corresponding to interaction score >0.9. The red- and blue-colored nodes signify up- and downregulated proteins, respectively. **(B)** Top-scoring PPI module consisting 19 nodes and 171 edges, where the adjacent edges of the *RPS3* gene with the highest degree (=25) is highlighted in red. PPI, protein–protein interaction.

A total of 606 genes comprising all the pathways and GO terms $\left(p-value<0.01\right.$ ) were inputted into the STRING database. The PPI network constructed using Cytoscape software included 302 nodes, and 688 edges are shown in Figure 3A. A total of 302 genes out of 1,120 TIG-DEGs participated in the PPI network at the selected highest confidence level, i.e., > 0.9. Moreover, Molecular Complex Detection (MCODE) revealed that the most highly significant cluster with a *top score = 18* was selected amongst all the 17 identified clusters, consisting 19 nodes and 171 edges (Figure 3B). Essential centrality measures such as node degree, betweenness, closeness, average shortest path length, clustering coefficient, radiality, and topological coefficient of the PPI network are provided in Supplementary Table S4. Based on the centrality measures, the significant hub gene RPS3 (ribosomal protein subunit 3, degree = 25), with the highest number of interacting partners, was identified.

### 3.5 Virtual screening of ligands

The PubChem library was searched with the keyword “COVID-19,” resulting in 1,709 compounds. The compounds were filtered out through three filtering processes (Martín-Sánchez et al., 2023): database filters (Ali et al., 2020), pharmacokinetic properties, and (Morse et al., 2020) toxicological properties. Through database filtering, only 886 candidates were selected that have a molecular weight between 100 and 500 g/mol, rotatable bond count between 0 and 7, h-bond acceptor count between 0 and 10, polar area between 0 and 150 Å^2^, and XlogP between −6.3 and 5. Government organizations, research and development, journal publishers, and NIH initiatives were selected as the data source category. The SwissADME webserver was accessed to calculate pharmacokinetic parameters. After the pharmacokinetic filtration process, 42 compounds that show zero violations of Lipinski’s rule, zero lead likeness violations, and high gastrointestinal (GI) were selected. To calculate toxicological parameters, the OSIRIS Property Explorer was used to predict the toxicity risks and drug scores of the 42 compounds. The program evaluated the risks of different side effects, such as mutagenic, irritant, tumorigenic, reproductive effects, and drug-related properties. Furthermore, the overall drug score was calculated by summing up various parameters such as cLogP, solubility (logS), molecular weight, drug-likeness, and toxicity risk. The final filtration process resulted in 28 compounds showing no toxicity risks and drug score values between 0.40 and 1.00, making it a good library for further docking studies (Table 3).

**TABLE 3:** Physiochemical properties of 28 selected drugs calculated after filtering thrice database filter, pharmacokinetic filter, and toxicological filter. The following drugs show no toxicity risks (mutagenicity, tumorigenic, irritant, and reproductive effective).

S.no.	PubChemID	Compound Name	Chemical formula	Osiris Property Explorer					Swiss ADME webserver			
				*MW*	*cLogP*	*Solubility*	*Drug-likeness*	*Drug score*	*TPSA*	*Lipinski*	*Bioavailability*	*Synthetic*
1	3365	Fluconazole	C_13_H_12_F_2_N_6_O	306.27	−0.11	−2.17	1.99	0.87	81.65	0	0.55	2.45
2	4236	Modafinil	C_15_H_15_NO_2_S	273.35	0.38	−3.84	0.52	0.69	79.37	0	0.55	2.83
3	4594	Omeprazole	C_17_H_19_N_3_O_3_S	345.42	2.09	−2.72	−0.51	0.6	96.31	0	0.55	3.58
4	5329	Sulfamethoxazole	C_10_H_11_N_3_O_3_S	253.28	0.44	−3.02	2.77	0.88	106.6	0	0.55	2.73
5	13730	2′-Deoxyadenosine	C_10_H_13_N_5_O_3_	251.24	−0.89	−2.72	−5.08	0.47	119.31	0	0.55	3.6
**6**	**27125**	**Estetrol**	**C**_**18**_ **H**_**24**_ **O**_**4**_	**304.38**	**2.17**	**−3.22**	**−0.61**	**0.58**	**80.92**	**0**	**0.55**	**3.99**
7	35370	Zidovudine	C_10_H_13_N_5_O_4_	267.24	−1.02	−1.44	2.12	0.91	134.07	0	0.55	3.93
8	73115	1-((2S,3R,4S,5S)-3-fluoro-4-hydroxy-5-(hydroxymethyl)tetrahydrofuran-2-yl)-5-methylpyrimidine-2,4(1H,3H)-dione	C_10_H_13_FN_2_O_5_	260.22	−1.4	−1.3	2.56	0.93	104.55	0	0.55	3.95
9	446541	Mycophenolic acid	C_17_H_20_O_6_	320.34	2.3	−3.01	−0.61	0.58	93.06	0	0.56	3.02
10	2566008	1-[4-(5-Chlorothiophen-2-yl)sulfonylpiperazin-1-yl]ethanone	C_10_H_13_ClN_2_O_3_S_2_	308.8	1.67	−1.28	5.94	0.93	94.31	0	0.55	3.08
11	3759658	1-[4-(Thiophene-2-sulfonyl)-piperazin-1-yl]-ethanone	C_10_H_14_N_2_O_3_S_2_	274.36	0.86	−0.52	6.03	0.95	94.31	0	0.55	2.88
12	3803220	2-(4-Acetylpiperazin-1-yl)sulfonylbenzonitrile	C_13_H_15_N_3_O_3_S	293.34	0.7	−1.38	1.42	0.85	89.86	0	0.55	2.62
**13**	**5281605**	**Baicalein**	**C**_**15**_ **H**_**10**_ **O**_**5**_	**270.24**	**2.34**	**−2.86**	**0.75**	**0.75**	**90.9**	**0**	**0.55**	**3.02**
14	5360515	Naltrexone	C_20_H_23_NO_4_	341.4	1.58	−3.19	4.67	0.85	70	0	0.55	4.72
15	6453528	Sulodexide	C_12_H_17_N_5_O_4_	295.29	−0.99	−2.44	−0.88	0.6	114.55	0	0.55	4.14
16	9926791	Tofacitinib	C_16_H_20_N_6_O	312.37	1.19	−3.59	−2.54	0.46	88.91	0	0.55	3.26
17	15277004	5′-Thiothymidine	C_10_H_14_N_2_O_4_S	258.29	−0.39	−2.28	−0.51	0.65	123.12	0	0.55	3.71
**18**	**23634441**	**Vadadustat**	**C**_**14**_ **H**_**11**_ **ClN**_**2**_ **O**_**4**_	**306.7**	**1.2**	**−3.15**	**−3.53**	**0.45**	**99.52**	**0**	**0.56**	**2.31**
19	25126798	Ruxolitinib	C_17_H_18_N_6_	306.37	1.68	−3.92	−6.97	0.41	83.18	0	0.55	3.16
20	25271624	Adrafinil, (R)-	C_15_H_15_NO_3_S	289.35	0.27	−4.04	0.85	0.7	85.61	0	0.55	3.21
21	27646027	1-acetyl-N-(6-methoxypyridin-3-yl)piperidine-4-carboxamide	C_14_H_19_N_3_O_3_	277.32	0.99	−1.93	5.77	0.94	71.53	0	0.55	2.37
22	47110626	1-acetyl-N-(2-hydroxyphenyl)piperidine-4-carboxamide	C_14_H_18_N_2_O_3_	262.3	1.36	−1.88	6.08	0.94	69.64	0	0.55	1.75
23	47289091	N-[3-(thiomorpholine-4-carbonyl)phenyl]acetamide	C_13_H_16_N_2_O_2_S	264.34	1.44	−2.35	3.05	0.91	74.71	0	0.55	2.01
24	61865966	3-{[(3-Methyl-1,2,4-oxadiazol-5-yl)methyl]carbamoyl}benzoic acid	C_12_H_11_N_3_O_4_	261.23	0.41	−1.34	−0.92	0.61	105.32	0	0.56	2.37
25	135398593	2′-Deoxyinosine	C_10_H_12_N_4_O_4_	252.23	−1.41	−2.02	3.04	0.93	113.26	0	0.55	3.51
26	145998218	1-(4-Methylpiperazin-1-yl)-2-(1H-pyrrolo [2,3-b]pyridin-3-yl)ethan-1-one	C_14_H_18_N_4_O	258.32	0.76	−1.37	9.16	0.95	52.23	0	0.55	2.12
27	146019222	Nalpha-acetyl-N-(3-bromoprop-2-yn-1-yl)-L-tyrosinamide	C_14_H_15_BrN_2_O_3_	339.18	1.08	−2.93	−3.14	0.46	78.43	0	0.55	2.74
28	146037571	N-(3-{[(2R)-4-oxoazetidin-2-yl]oxy}phenyl)-2-(pyrimidin-5-yl)acetamide	C_15_H_14_N_4_O_3_	298.3	0.67	−2.31	1.63	0.85	93.21	0	0.55	2.95

Abbreviations, MW, molecular weight (g/mol); cLogP, calculated Log P; TPSA, topological polar surface area (in Å).

Bold values represent the final compounds selected in the result section.

### 3.6 Molecular docking

**FIGURE 4:**
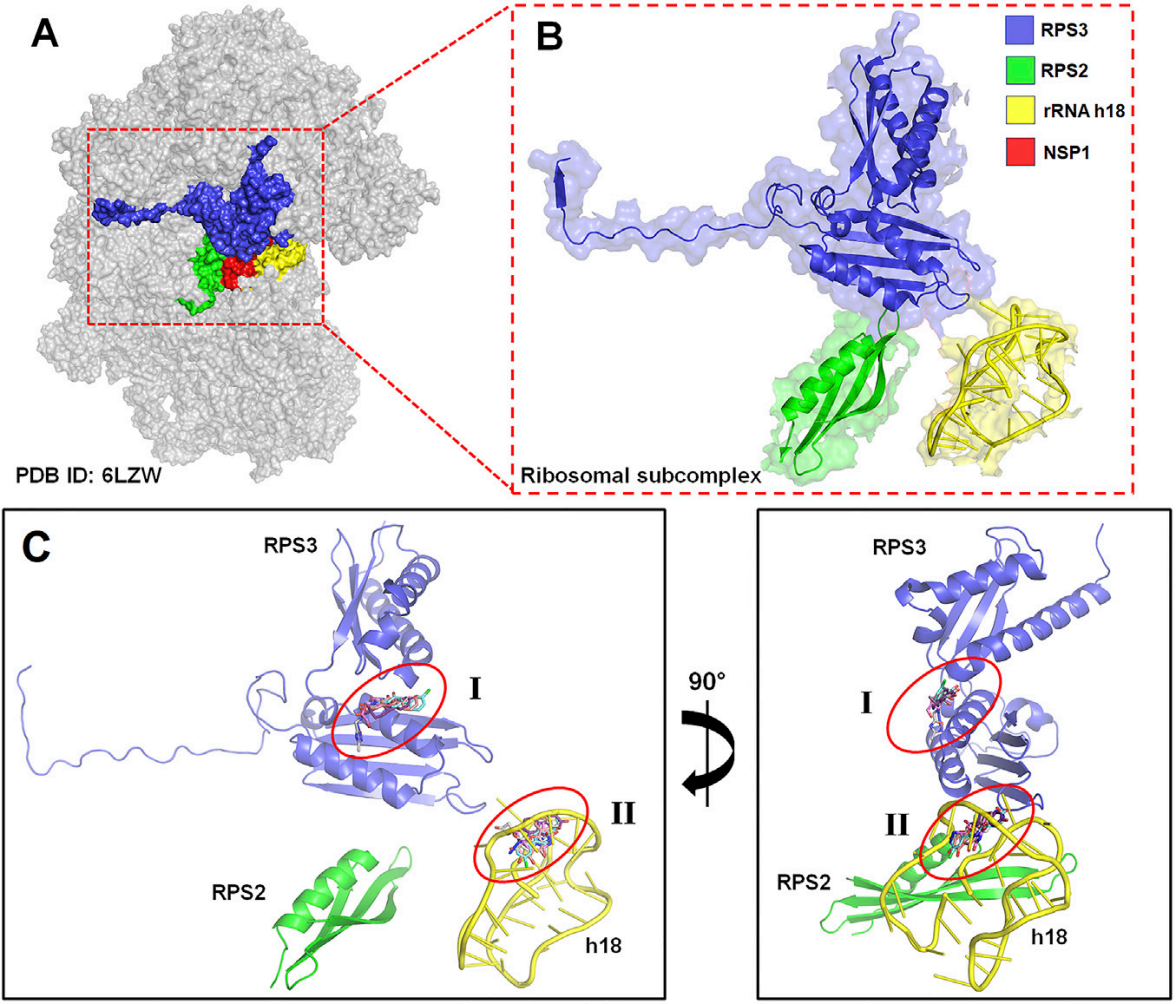
**(A)** Three-dimensional EM structure of the human 40S ribosomal subunit (PDB ID: 6ZLW), bounded to the ribosomal subcomplex and SARS-CoV-2 NSP1 protein (in red). **(B)** Magnified figure in cartoon representation of the ribosomal subcomplex comprised RPS3 (in blue), RPS2 (in green), and the phosphate backbone of rRNA helix 18 (h18) (in yellow) of 40S ribosomal protein. **(C)** Schematic representation of the two possible ligand-binding site pockets in the ribosomal subcomplex **(I)** in the center of RPS3 and **(II)** the head of rRNA h18 identified through blind docking.

**FIGURE 5:**
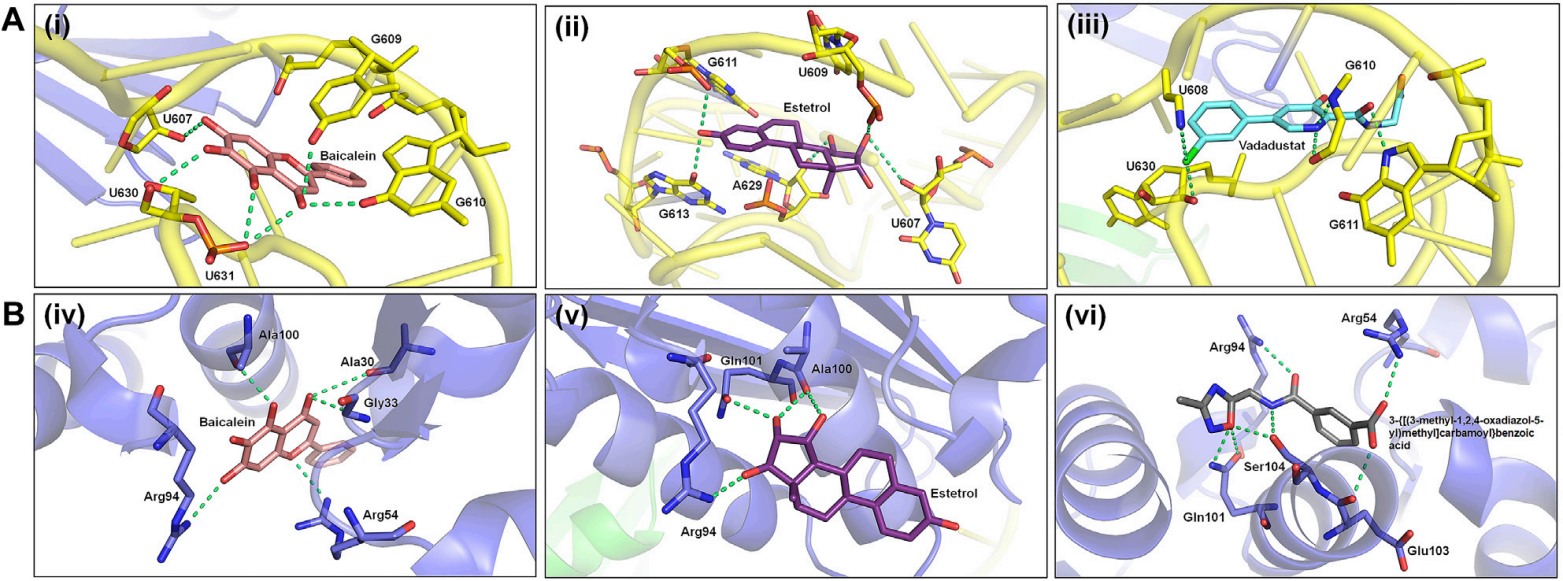
**(A)** Hydrogen bond interactions of baicalein, estetrol, and vadadustat (docked conformations) with the nucleotides of the phosphate backbone of h18. **(B)** Hydrogen bond interactions of baicalein, estetrol, and 3-{[(3-methyl-1,2,4-oxadiazol-5-yl)methyl]carbamoyl}benzoic acid (docked conformations) with active site residues of the RPS3 protein.

In this study, blind docking was performed since the subcomplex receptor of ribosomal protein has no ligand-binding site (Figures 4A, B). The rotatable bonds of the 28 ligands were kept flexible, while the subcomplex macromolecule was adopted as a rigid structure. The analysis of the molecular docking results shows that most of the ligands screened bind into two active sites: (I) the center of RPS3 protein and (II) the head of rRNA helix 18 (Figure 4C). The top three binding poses with the best binding energies at each ligand-binding site are given in Table 4. The best binding energies of compound baicalein, vadadustat, and estetrol are observed at the head of the rRNA helix 18 site with binding energies −8.24, −8.01, and −7.98 kcal/mol, respectively. Similarly, baicalein, estetrol, and 3-{[(3-methyl-1,2,4-oxadiazol-5-yl)methyl]carbamoyl}benzoic acid show the best binding energies at the center of RPS3 protein with binding energies −7.54, −7.33, and −7.24 kcal/mol, respectively. Baicalein has been shown to have the highest binding pose for both ligand-binding sites. All the compounds form hydrogen bonds with the rRNA helix18 site that contains the following nucleotides: U607, C608, U609, G610, G611, G613, U630, A629, and U631, whereas the RPS3-binding site contains the following residues: Ala30, Gly33, Arg54, Arg94, Ala100, Gln101, Glu103, and Ser104 (Table 4). The interactions of baicalein, estetrol, and vadadustat (rRNA h18) are shown in Figure 5A, whereas the interactions of baicalein, estetrol, and 3-{[(3-methyl-1,2,4-oxadiazol-5-yl)methyl]carbamoyl}benzoic acid (RPS3 protein) are shown in Figure 5B. In Figure 5A, (i) baicalein binds in the region of rRNA h18 through h-bond interactions with U607, G609, G610, U630, and U631; (ii) estetrol shows h-bond interactions with U607, U609, G611, G613, and A629; and (iii) vadadustat forms h-bond interactions with U608, G610, G611, and U630. Meanwhile, in Figure 5B, (iv) baicalein binds in the region of RPS3 protein through h-bond interactions with residues Ala30, Gly33, Arg54, Arg94, and Ala100; (v) estetrol forms h-bond interactions with residues Arg94, Ala100, and Gln101; and (vi) 3-{[(3-methyl-1,2,4-oxadiazol-5-yl)methyl]carbamoyl}benzoic acid forms h-bond with residues Arg54, Arg94, Gln101, Glu103, and Ser104.

**TABLE 4:** Top three docking pose prediction results for each ligand-binding sites: (I) the head of rRNA helix 18 and (II) the center of RPS3 protein.

Ligand Binding site	S. No.	Compound	PubChem ID	Binding Affinity (kcal/mol)	H-bond interactions (≤3.50 Å)
I. rRNA helix 18 (h18)	1	Baicalein	5281605	−8.24	U607^O3’^ (3.12), U607^O2’^ (3.09), U630^O3’^ (3.25), U630^O2’^ (3.27), U630^O3’^ (3.07), U631^OP1^ (2.85), U609^O4^ (3.33), U631^OP1^ (3.30), G610^OP1^ (2.90)
	2	Vadadustat	23634441	−8.01	G611^N7^(3.00), G610^N7^(3.07), C608^N4^(3.57), U630^O3’^(2.93)
	3	Estetrol	27125	−7.98	G613^O6^ (3.14), G611^OP2^ (3.19), A629^O2’^ (3.33), U609^OP2^ (3.10), U607^O2’^ (2.92)
II. RPS3 protein	1	Baicalein	5281605	−7.54	Arg54^NH2^(3.29), Arg94^NH1^(3.22), Ala100^OB^(2.95), Gly33^NB^(3.60), Ala30^OB^(3.55)
	2	Estetrol	27125	−7.33	Arg94^NH1^(2.68), Gln101^OE1^(3.04), Ala100^OB^(2.70), Ala100^OB^(3.31)
	3	3-{[(3-methyl-1,2,4-oxadiazol-5-yl)methyl]carbamoyl}benzoic acid	61865966	−7.24	Ser104^OG^(2.88), Gln101^OE1^(3.59), Gln101^NE2^(2.87), Arg54^NH2^(3.04), Glu103^OB^(3.05), Ser104^OG^(3.27), Arg94^NH1^(3.00)

## 4 Discussion

COVID-19 has highlighted several therapeutic gaps in healthcare. One of the most significant gaps is the lack of effective antiviral drugs to treat the disease. Although some drugs have been repurposed to treat COVID-19, such as remdesivir, their efficacy is limited, and they are not a cure for the disease. Another gap is the lack of effective treatments for severe cases of COVID-19. While some patients recover from the disease with supportive care, there are still many who die from the disease. There is an urgent need for more effective treatments to reduce the mortality rate of COVID-19.

The present study used bioinformatics tools to explore the structural similarities between human host proteins and SARS-CoV-2 proteins and their interacting partners. This analysis led to the identification of an impressive number of 6,116 human proteins and 19,051 unique interacting partners of SARS-CoV-2 proteins. Further analyses of the target interactors of hCoV-2 proteins, combined with the differentially expressed genes (DEGs) of COVID-19 patients, revealed a total of 1,120 TIG-DEGs that participated in Gene Ontology and pathway enrichment analyses. Remarkably, the most significant pathways and GO terms identified were related to coronavirus disease, the cellular macromolecule biosynthetic process, RNA binding, and ribosome. Subsequently, the PPI network analysis identified *RPS3* as the hub gene in a cluster of 19 nodes and 171 edges.

It should be noted that the approach of identifying host proteins structurally similar to viral proteins has been used before, as evidenced by Gordon et al. (2020). Nonetheless, the current study identified a much higher number of host proteins and interacting partners, due to the utilization of a different method of merging structurally similar host proteins and removing duplicates. Similarly, the approach of comparing the target interactors of hCoV-2 proteins with the DEGs of COVID-19 patients has been used before, as demonstrated by Blanco-Melo et al. (2020). Despite this, the present study identified a larger number of TIG-DEGs, indicating greater level of sensitivity in the methodology used.

The PPI network analysis of TIG-DEGs using Cytoscape software is a well-established method in systems biology that allows the identification of hub genes and protein complexes. The identification of *RPS3* as a hub gene in the present study is particularly noteworthy, as it has also been identified by Prasad et al. (2021) as a hub gene in their PPI network analysis of SARS-CoV-2 interactors. The current study and findings from these two earlier studies suggest that RPS3 could be a potential therapeutic target for COVID-19.

Last, filtering potential drug candidates from an extensive library of compounds based on various pharmacokinetic and toxicological properties is a conventional strategy in drug discovery (Reyaz et al., 2021; Sultan et al., 2021; Sultan et al., 2022). This study identified three compounds—baicalein, vadadustat, and estetrol—with promising properties, such as binding affinity and residual interaction with RPS3, for COVID-19 treatment. These compounds could be further tested *in vitro* and *in vivo* for their efficacy. Although different research groups have tried to investigate that baicalein may act as an anti-SARS-CoV-2 drug through *in vitro* analysis, its safety and efficacy in SARS-CoV-2-infected transgenic animals have not been studied yet (Huang et al., 2020; Su et al., 2020; Liu H. et al., 2021; Dinda et al., 2023). However, Song et al. (2021) investigated the therapeutic effect of baicalein against SARS-CoV-2 both *in vivo* and *in vitro* (Song et al., 2021). Further experimental studies are recommended to prove that baicalein may act as an effective anti-COVID-19 molecule.

In summary, this study’s bioinformatics analysis provides novel insights into the biology of SARS-CoV-2 infection and potential therapeutic targets. Although some of the methodologies used have been used before, this study’s findings highlight the importance of leveraging multiple bioinformatics tools to identify host proteins, interacting partners, and potential drug candidates. Nonetheless, further experimental validation is necessary to confirm this study’s findings and facilitate the translation of these findings into clinically applicable interventions. This study has some strengths and some limitations. One of the major strengths of this study is the comprehensive and systematic approach toward identifying the host proteins that interact with SARS-CoV-2 proteins and their potential roles in COVID-19 pathogenesis. This study used multiple databases and tools to identify the interactors and DEGs in COVID-19 patients. This approach provides a more comprehensive understanding of the host–virus interactions and their potential roles in the disease pathogenesis. This study also identified significant pathways and GO terms that could provide potential targets for drug development. However, this study also has some limitations that need to be addressed. This study relied solely on *in silico* analysis and did not validate the identified DEGs or hub genes through experimental studies. Additionally, this study used only one mRNA expression profile dataset, which may not be sufficient to capture the diversity of gene expression patterns in COVID-19 patients. This study also did not investigate the functional roles of the identified hub gene containing RPS3, which limits the understanding of its potential importance in COVID-19 pathogenesis.

## 5 Conclusion

In conclusion, the current study provides a comprehensive and systematic approach to identifying potential therapeutic targets for COVID-19 by exploring the structural similarity of human host proteins to SARS-CoV-2 proteins and investigating their interactions and pathways. This study identified 19,051 unique interactors for SARS-CoV-2 proteins, 1,120 differentially expressed genes, and a significant hub gene containing RPS3. The screening of the PubChem library identified three compounds, baicalein, vadadustat, and estetrol, with the best binding energies and residual interaction with the target molecule. Overall, current findings suggest that exploring the human host proteins’ interactions and pathways can provide valuable insights for developing effective treatments for COVID-19.

## Data Availability

The dataset used in our study is publicly available and can be downloaded from the National Center for Biotechnology Information Gene Expression Omnibus under accession number GSE164805 (https://www.ncbi.nlm.nih.gov/geo/query/acc.cgi?acc=GSE164805).

## Abbreviations

COVID-19, coronavirus disease 2019; h18, rRNA helix 18; hCoV-2, human proteins structurally similar to SARS-CoV-2; HIV, human immunodeficiency virus; HPs, human proteins; HV-PPIs, host–virus protein–protein interactions; NSP, non-structural protein; ORF, open reading frame; RPS2, ribosomal protein subunit 2; RPS3, ribosomal protein subunit 3; SARS-CoV-2, severe acute respiratory syndrome coronavirus 2; T_hCoV-2, targets of hCoV-2; TIG-DEGs, targets and infected group DEGs; and ZIKV, Zika virus.
